# Programming new geometry restraints: parallelity of atomic groups

**DOI:** 10.1107/S1600576715010432

**Published:** 2015-07-08

**Authors:** Oleg V. Sobolev, Pavel V. Afonine, Paul D. Adams, Alexandre Urzhumtsev

**Affiliations:** a Lawrence Berkeley National Laboratory, One Cyclotron Road, MS64R0121, Berkeley, CA 94720, USA; b Department of Bioengineering, University of California Berkeley, Berkeley, CA 94720, USA; c Centre for Integrative Biology, IGBMC, CNRS-INSERM-UdS, 1 rue Laurent Fries, BP 10142, 67404 Illkirch, France; d Département de Physique, Faculté des Sciences et des Technologies, Université de Lorraine, 54506 Vandoeuvre-lès-Nancy, France

**Keywords:** restraints, atomic model refinement, parallel planes, *cctbx*, *PHENIX*, gradient calculation

## Abstract

Improvements in structural biology methods, in particular crystallography and cryo-electron microscopy, have created an increased demand for the refinement of atomic models against low-resolution experimental data. One way to compensate for the lack of high-resolution experimental data is to use *a priori* information about model geometry that can be utilized in refinement in the form of stereochemical restraints or constraints. Here, the definition and calculation of the restraints that can be imposed on planar atomic groups, in particular the angle between such groups, are described. Detailed derivations of the restraint targets and their gradients are provided so that they can be readily implemented in other contexts. Practical implementations of the restraints, and of associated data structures, in the *Computational Crystallography Toolbox* (*cctbx*) are presented.

## Abstract 2

Details are described of the calculation of new parallelity restraints recently introduced in *cctbx* and *PHENIX*.

## 1. Introduction   

The restrained refinement of atomic models is performed through minimization of a target function. First introduced in the macromolecular crystallography field by Konnert (1976 ▸) and Jack & Levitt (1978 ▸), this target is a weighted sum of several terms including 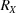 describing the model fit to the experimental data:
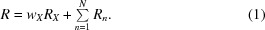
In the case of refinement of atomic coordinates, the terms 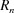 describe the model geometry and are known as geometric or stereochemical restraints.

At low resolution there may be many models that may fit the experimental data equally well, but not all of these models may be chemically reasonable. This is why additional information needs to be used to restrict the models to those that conform to expectations about the correct chemical structure. These expectations may originate from various sources, such as small-molecule crystallographic studies, high-quality macromolecular structures or quantum chemistry calculations, forming the basis for libraries of *a priori* knowledge. At lower resolution, there are fewer experimental data available and therefore a larger number of restraints are required to ensure a high-quality refined model. Therefore, low-resolution structural studies, which have become more and more frequent, require additional restraints (see, for example, Smart *et al.*, 2012 ▸; Headd *et al.*, 2012 ▸; Brown *et al.*, 2015 ▸) to complement the traditionally used restraints, such as bond lengths, bond angles, dihedral angles, non-bonded interactions, and planarity and chirality restraints.

As an example, nucleic acids have a number of base pairs, some of which are parallel or nearly parallel to each other. This knowledge may serve as a restraint that can be applied to the refinement of such models. Adding a new restraint into the refinement target means developing a corresponding algorithm to calculate its value, as well as an algorithm to calculate the gradient of this restraint function. We use the example of the parallelity restraint, implemented in *cctbx* (Grosse-Kunstleve *et al.*, 2002 ▸), to illustrate the general approach of adding new restraints in *cctbx* and *PHENIX* (Adams *et al.*, 2010 ▸) and also to reinforce the general principles of crystallographic refinement programs. This article describes the algorithms in a ready-to-program manner, with sufficient mathematical and computational details to serve a didactic purpose as well. Practical examples of the application of this new restraint function to model refinement will be presented elsewhere.

Each individual target in equation (1) is the sum of a large number of identical terms, each depending on a small number of parameters. For example, a diffraction target usually depends on a structure factor amplitude or the parameters of its phase distribution, a density target depends on the density values in a few grid points around a particular atom (see, for example, Afonine & Urzhumtsev, 2004 ▸, and references therein), a geometric restraint depends on the coordinates of a few atoms involved in particular restraint, *etc*. Such a composition of the target function (1) is very important for several reasons. First, it allows the manipulation of each kind of restraint, *e.g.* selection of the atoms involved or an update of the function. Second, each restraint is relatively simple and easy to program. Third, each restraint can be weighted individually. Finally, the gradient of the crystallographic target used for the minimization can be calculated as a sum of gradients from the individual terms.

Several general points are important to note when designing refinement programs (Lunin & Urzhumtsev, 1985 ▸):

(i) Refinement targets may be functions of the atomic parameters and values of electron density (or structure factors), in combination with the restraints and/or constraints imposed on the atomic model. Each component of the overall target is a relatively simple function when it is expressed through the parameters of the appropriate type.

(ii) These parameters are calculated from each other as a chain of consecutive transitions.

(iii) The gradient calculations are performed step by step using the chain rule, inverting the chain of steps used to calculate the target (Baur & Strassen, 1983 ▸; Kim *et al.*, 1984 ▸). This procedure results in exact gradient values that require approximately the same amount of time to calculate as a single value of the target, and this is independent of the number of parameters to be refined.

(iv) The gradient calculation does not require an analytical function; it may be any algorithm that provides a value for the corresponding target. At each refinement step, the target or gradient calculation is a local operation. It takes the numerical values obtained at the previous step, calculates the result of the step and passes the numerical values to the next step. Such a scheme allows one to avoid complicated mathematical equations connecting the final target or gradient value with the initial model parameters.

In order to introduce a parallelity restraint into refinement, the considerations stated above require that we propose an algorithm to calculate a numerical value describing by how much two atomic groups that form pseudo-planes (approximately belong to two planes) are not parallel to each other. This algorithm should be numerically robust enough to handle a broad range of model parameters, from good (towards the end of refinement) to distorted (at the beginning of refinement).

## 2. Possible parallelity restraints and their gradient   

### 2.1. Definition of the ‘best plane’   

Let us consider two pseudo-planar atomic groups (groups that should be planar but may not currently be so), *m* = 1, 2, containing atoms defined by their Cartesian coordinates, **r**_*k*,*m*_ = (*x*_*k*,*m*_, *y*_*k*,*m*_, *z*_*k*,*m*_), *k* = 1, …, *K_m_*, in the same coordinate system. For each such group we need to define the corresponding ‘best plane’. A plane is defined by a vector **N**_*m*_ normal to it and by a point **C**_*m*_ that belongs to it. For a given atomic group, the plane can be characterized by the sum of the squared deviations of the atoms from it (Urzhumtsev, 1991 ▸; Blanc & Paciorek, 2001 ▸; Brown *et al.*, 2015 ▸), and these deviations may be weighted if desired. The ‘best plane’ is the one that corresponds to the minimum of this value (**N**_*m*_ and **C**_*m*_ are variables),
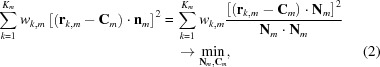
where *w*_*k*,*m*_ is a weight (*e.g.* a unit value or an atomic mass) for the contribution of an atom with coordinates **r**_*k*,*m*_ to the target. Here and below, a centred dot ‘·’ represents the scalar (dot) product of two vectors, and 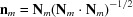 is the normalized vector **N**_*m*_.

Two facts are useful (see, for example, Urzhumtsev, 1991 ▸). First, the minimum of equation (2) is reached when **C**_*m*_ is the weighted geometric center of the group
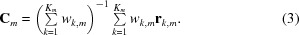
Second, if for a given atomic group we calculate the minimal eigenvalue of its inertia matrix (see §3.2), the corresponding eigenvector is normal to the best plane defined by condition (2) and therefore it can be taken as **N**_*m*_.

The angle between two atomic groups can be described by the angle θ between two vectors normal to the best planes for the corresponding groups. This angle varies between the limits 0 ≤ θ ≤ π/2. This angle is expected to be zero if we require the planes to be parallel, which is the most frequent restraint of this kind. We therefore need to design a function which reaches its minimum when θ = 0. Note that there are multiple functions which could be constructed such that θ_0_ = 0 is a minimum. Also, the particular restraint function needs to describe the stereochemical knowledge near the point of interest (minimum) and have a desired behavior far from it. Additionally, the computation of the restraint and its derivatives must have no numerical irregularities. Below, we analyze some possible restraints that describe the angle between two planar groups, noting that different restraints can be used in different circumstances.

### 2.2. Analysis of various possible parallelity targets   

The computationally simplest target to restrain the angle between normal vectors is
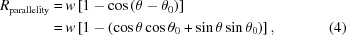
where θ = θ(**n**_1_, **n**_2_) is the angle between two unit vectors **n**_1_ and **n**_2_, θ_0_ is the target angle, and *w* is the individual weight factor for a particular pair of planes. By default, *phenix.refine* (Afonine *et al.*, 2012 ▸) uses the form *w* = 1/σ^2^, where *σ* is the root mean-square deviation from the ideal value obtained by some means. Introducing the angle θ_0_ in equation (4) will force the planes to form the prescribed angle between them. For example, as suggested by Brown *et al.* (2015 ▸), a restraint with θ_0_ = π/2 is important when working with aromatic groups. For simplicity, we continue to call this restraint ‘parallelity’, even though this is not completely appropriate. We note that cosθ = **n**_1_·**n**_2_ and sinθ = ||**n**_1_ × **n**_2_||.

For small deviations from θ_0_, restraint (4) behaves as the quadratic function 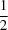 *w*(θ − θ_0_)^2^. However, using the quadratic function of the angle itself [see in particular equation (14) of Brown *et al.* (2015 ▸)] requires its derivatives, *i.e.* the derivative of arccos(**n**_1_·**n**_2_), which is undefined for θ = θ_0_. Also, its calculation is computationally unstable for θ ≃ θ_0_. For this reason, we have excluded this kind of target from our consideration.

Also, a seemingly plausible restraint function that one may wish to avoid is
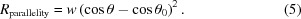
This function is easy to calculate but it has very different behavior across different ranges of the parameter θ_0_. For example, it is similar to a second-order polynomial near θ_0_ = π/2 and to a fourth-order polynomial near θ_0_ = 0. Such heterogeneity is generally disadvantageous for minimization.

Another known target for the parallelity restraint is
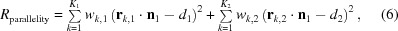
[equation (13) of Brown *et al.* (2015 ▸); the notation of the present article is used]. It imposes a rigid condition on the parallelity of two planes, **n**_2_ = **n**_1_, simultaneously with planarity of the corresponding groups. This constraint-type condition may be useful, for example, to simplify the parameterization of the model, but in practice nucleic acid bases may bend from one base pair to another, or they may be twisted inside a given base pair. As a consequence, it is more convenient to have planarity and parallelity restraints defined separately, allowing for more flexibility if desired, in particular allowing the interplanar angle to vary from one planar group to another.

**Figure 1:**
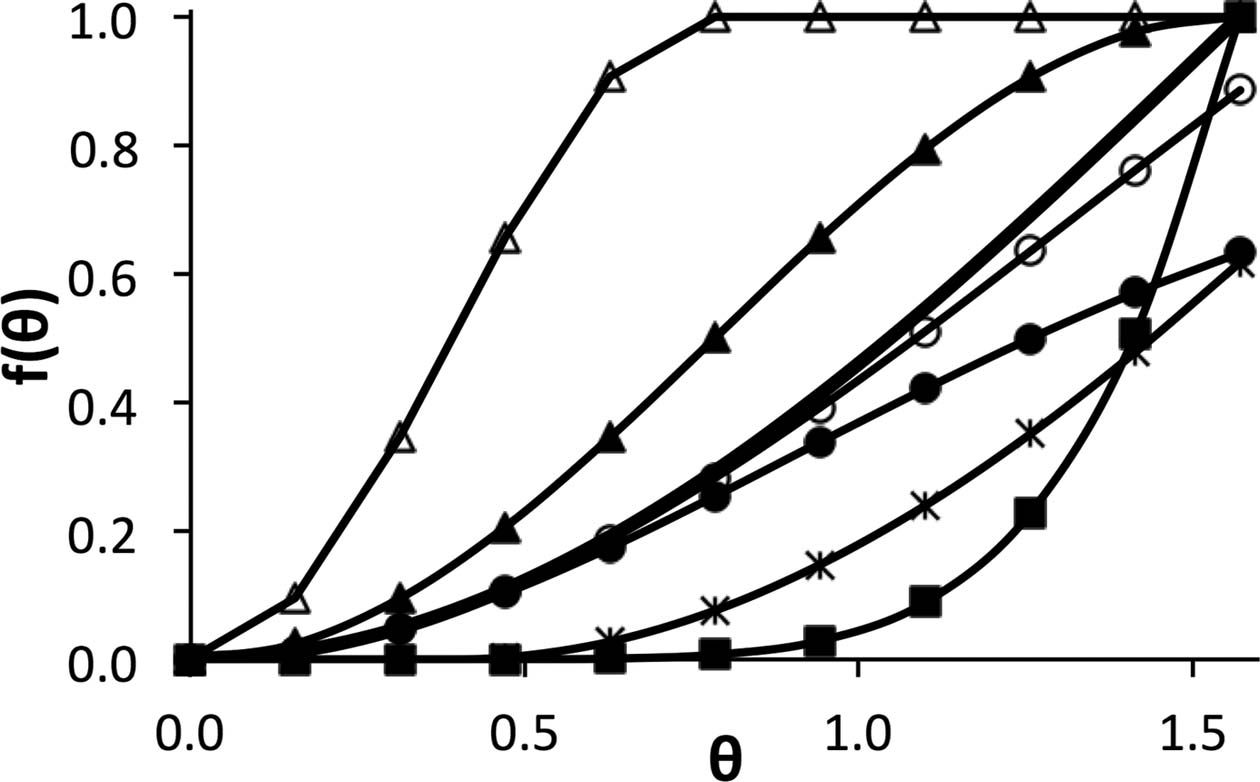
Examples of various parallelity targets, θ_0_ = 0. Heavy line with no markers: *f*(θ) = 1 − cosθ, equation (4). Line with filled triangles: *f*(θ) = 1 − cos2θ, equation (8). Line with open triangles: *f*(θ) = 1 − cos4θ for θ ≤ π/4, equation (9). Lines with black (Ω = 1) and open (Ω = 2) circles: *f*(θ) = Ω^2^{1 − exp[(cosθ − 1)/Ω^2^]}, equation (7). Line with filled squares: *f*(θ) = (1 − cosθ)^4^, equation (10). Line with asterisks: *f*(θ), slack function, equation (11).

When the function is far from the minimum (*e.g.* when the current structure is distorted with respect to the restraints), one may wish that the target be relatively insensitive to the variation in the angle θ. This can be achieved by using various kinds of ‘top-out’ functions (Dennis & Welsch, 1987 ▸; Murshudov *et al.*, 2011 ▸; Smart *et al.*, 2012 ▸; Headd *et al.*, 2012 ▸). A direct application of this idea results in
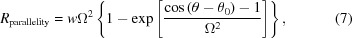
where Ω is a constant (Fig. 1 ▸). Using (θ − θ_0_)^2^ or (cosθ − cosθ_0_)^2^ in equation (7) instead of cos(θ − θ_0_) − 1 is not desirable for the reasons discussed above. In fact, we can use directly a sigmoid form of the cosine function and introduce the ‘top-out type’ targets in a simpler form:
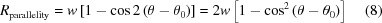
or
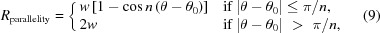
where *n* > 2. These functions (Fig. 1 ▸) have harmonic behavior when θ ≃ θ_0_, while for large values of the argument they are constant [equation (9)] or nearly constant [equation (8)].

A further useful modification of target function (4) can be achieved by introducing the ‘slack’ parameter (square well-like potential), which scores small deviations from the target value equally. This can be achieved in a ‘soft’ way (Fig. 1 ▸) as 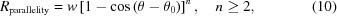
or following explicitly the slack definition (Fig. 1 ▸) as
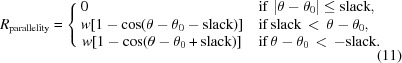



### 2.3. Practical calculation of the parallelity targets and their gradient   

All the restraint functions mentioned above are similar in the sense that they require calculation of the normal vectors to the optimal planes of the atomic groups; then their dot product and finally a vector product of these vectors are calculated to obtain the target value.

**Figure 2:**
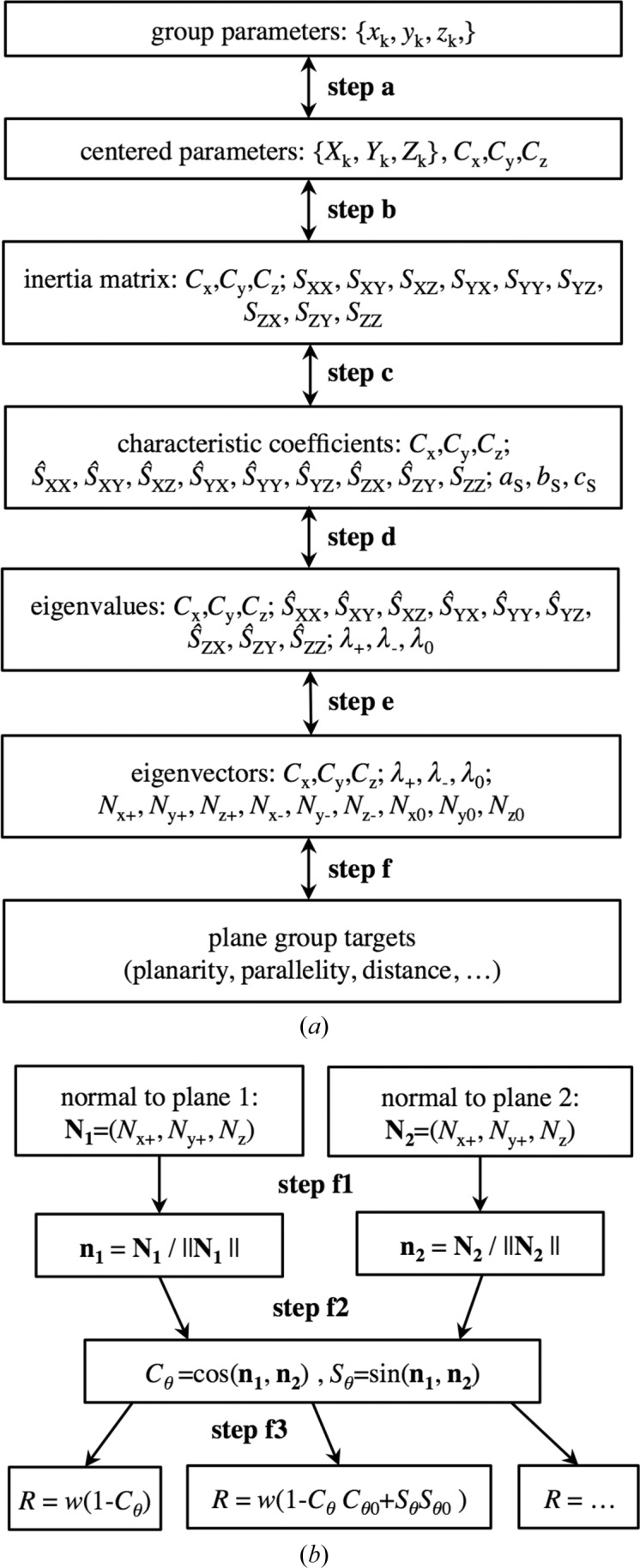
The overall calculation scheme. (*a*) The steps used to calculate parameters common to all targets. The letters *a*–*f* from top to bottom refer to the steps for calculating the target described in §3. The same steps in the direction from bottom to top refer to the steps for calculating the derivatives described in §4. The steps are applied independently to each atomic group required to be planar. (*b*) The particular parallelity targets that can be constructed on the basis of the calculated parameters of an atomic group.

A number of other restraints, such as group planarity or the distance between parallel groups, are naturally expressed through the same types of parameters used for the parallelity restraint (Appendix *A* [App appa]). These parameters are the center of the atomic group, and the eigenvalues and eigenvectors of its inertia matrix (generally speaking, all three eigenvalues and all three eigenvectors may be required); a particular target may use only some of the parameters mentioned. Thus, the calculation of these parameters from the initial set of atomic coordinates is common to all such restraints on planar groups (Fig. 2 ▸). For this reason, in §3 we provide details of these common steps.

Assuming we have a function that we can calculate numerically, the principle of efficient gradient calculation (Baur & Strassen, 1983 ▸; Kim *et al.*, 1984 ▸) suggests inverting the steps of the function’s calculation and obtaining the gradient step by step using the chain rule. This would result in the exact value of the gradient with respect to the original parameters, and this calculation will take no more than four times the CPU time required to calculate a single value of the function. This factor of four is invariant with respect to the number of parameters and the function type. In crystallographic practice, this factor is typically closer to one than to four (Lunin & Urzhumtsev, 1985 ▸). §4 illustrates this principle by calculating the gradient of the parallelity restraints. It is easy to see that the calculation of the gradient of other restraints for pseudo-plane groups differs only in the first step.

## 3. Planarity targets calculation   

This section describes the calculation of the target value for the suggested restraint. Fig. 2 ▸ shows the overall scheme, where the letters *a* to *f* refer to the corresponding steps described below.

### 3.1. Centered coordinate system (Step *a*)   

We start by calculating the atomic positions in the coordinate system with the origin shifted into the geometric center **C**_*m*_ of the group, equation (3). This operation is similar for all pseudo-plane groups, and in what follows we omit the group index *m* to simplify the formulae. The new parameters are the Cartesian coordinates (*X*_*k*_, *Y_k_*, *Z_k_*) of the atomic positions: 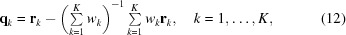
and the Cartesian coordinates (*C_x_*, *C_y_*, *C_z_*) of the weighted geometric center of the group.

### 3.2. Inertia matrix (Step *b*)   

For a given atomic group, its inertia matrix is
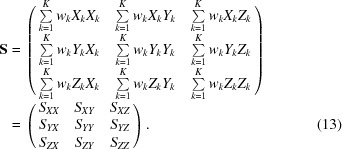
This matrix is symmetric, *S_XY_* = *S_YX_*, *S_YZ_* = *S_ZY_*, *S_ZX_* = *S_XZ_*, but for convenience in subsequent calculations we refer to all nine of its elements. Matrix (13) is positive semidefinite, having always three non-negative eigenvalues, 0 ≤ λ_+_ ≤ λ_−_ ≤ λ_0_, with the corresponding eigenvectors orthogonal to each other. The result of this step contains nine elements of matrix (13) and three coordinates of the center [equation (12)] taken from the previous step.

It is important to remember that at each step we calculate the *values* of the new parameters and do not keep their analytical expression.

### 3.3. Characteristic equation (Step *c*)   

The eigenvalues 0 ≤ λ_+_ ≤ λ_−_ ≤ λ_0_ of the inertia matrix (13) are the roots of the cubic characteristic equation
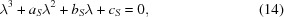
with the coefficients 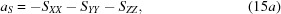


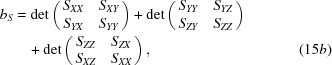


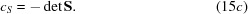
Further steps of the procedure use both the coefficients of the cubic equation (14) and the elements of the inertia matrix (13). To avoid confusion when calculating the gradients [see, for example, equation (18) below], it is more convenient to introduce new variables formally:
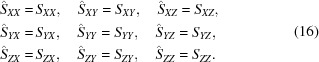
Thus, the new set of parameters contains 15 values: three coordinates (*C_x_*, *C_y_*, *C_z_*) of the center of the atomic group that are simply taken from the previous step, three equation coefficients *a_S_*, *b_S_*, *c_S_*, and nine elements of the inertia matrix 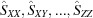.

### 3.4. Eigenvalues of the inertia matrix (Step *d*)   

Different algorithms may be applied to calculate the eigenvalues and choose the minimum of them; we use the one described by Urzhumtsev (1991 ▸), as it gives analytically the minimal real eigenvalue that is required for the planarity and parallelity restraints. Appendix *B* [App appb] gives an extended description in the notation of the present paper. The coordinates of the center of the atomic group [equation (12)] and the matrix elements [equation (16)] are not used at this step and pass to the next steps without change.

### 3.5. Eigenvectors of the inertia matrix (Step *e*)   

Different algorithms may be applied to calculate the eigenvectors **N** of the inertia matrix that are solutions of the matrix equation
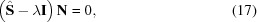
with the eigenvalues λ obtained in the previous step. Appendix *B* [App appb] gives technical details of the method used in our program. For an atomic group that is approximately planar, λ_+_ < λ_−_, the solution of equation (17) with λ = λ_+_ defines unambiguously a single direction **N**_+_ of the normal to the best plane.

Note that other restraints on the plane groups may require knowledge of other eigenvalues and/or eigenvectors (Appendix *A* [App appa]). Therefore, in the general case, the result of this step is a set of parameters fully describing a pseudo-planar atomic group, namely the three coordinates (*C_x_*, *C_y_*, *C_z_*) of its center, all three eigenvalues 0 ≤ λ_+_ ≤ λ_−_ ≤ λ_0_ and all three eigenvectors **N**_+_, **N**_−_, **N**_0_ of the inertia matrix.

### 3.6. Parallelity targets (Step *f*)   

To calculate a planarity restraint for two pseudo-planar atomic groups, each described by the Cartesian coordinates of the vector **N**_1_ = **N**_1+_ and **N**_2_ = **N**_2+_ normal to their respective best planes, we may need to follow several intermediate steps (Appendix *D* [App appd]). We start (Step *f*1) from normalization of the normal vectors, {**N**_1_, **N**_2_} 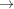 {**n**_1_, **n**_2_}. This normalization includes choosing the correct direction of the normalized vectors so that the value of the angle θ between the normal vectors is not larger than π/2. Once the normalized vectors are known, we calculate (Step *f*2) the cosine and sine of the angle θ between them, which we denote *C*_θ_ and *S*_θ_, respectively. Finally (Step *f*3), we calculate the value of the chosen target.

## 4. Gradient calculation   

### 4.1. Gradient with respect to the plane parameters   

We start by inverting the formulae to calculate the chosen target (Step *f*3 in Fig. 2 ▸) and obtain 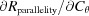 and 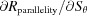 (Appendix *D* [App appd]).

Inverting the previous step (Step *f*2), we take as input the values of 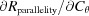 and 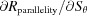 (and not their analytical expressions), whatever the target *R*_parallelity_ is. As a result, we obtain (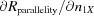, 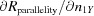, 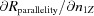) and (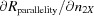, 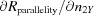, 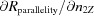). Finally, inverting the normalization (Step *f*1) we obtain (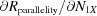, 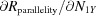, 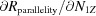) and (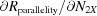, 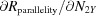, 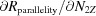).

Working with other restraints on plane groups, we can apply the same kind of procedure as Steps *f*3 to *f*1 above. For all these targets, we arrive at a set of partial derivatives of a target with respect to the following:

(i) The three eigenvalues for each pseudo-planar group.

(ii) The coordinates of the three eigenvectors for each pseudo-planar group.

(iii) The coordinates of the center of each pseudo-planar group.

For the parallelity restraints discussed above, all these derivatives are equal to zero except those for the coordinates of **N**_1+_ and **N**_2+_. Further steps of the gradient calculation use these partial derivatives as input data whatever the target is. These steps are common to all restraints and all atomic groups. Therefore, starting with the next section we omit the index used to indicate an atomic group and the index of a particular plane-group restraint for which all the partial derivatives above have been calculated. Partial derivatives with respect to the coordinates (*C_x_*, *C_y_*, *C_z_*) will not change until the last step.

### 4.2. Gradient with respect to the coefficients of the characteristic equation (Steps *e* and *d*)   

As mentioned in §§3.4 and 3.5, different algorithms exist for calculating the eigenvalues and eigenvectors of the inertia matrix from the coefficients of the characteristic equation (14). According to the main scheme, we invert these algorithms to calculate the corresponding derivatives. Appendix *C* [App appc] provides these for the particular method of calculation chosen in our program. The output of this step contains the partial derivatives of the target *R* with respect to the three coefficients *a_S_*, *b_S_*, *c_S_* of equation (14), to the nine elements 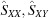, …, 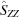 of equation (16), and to the three coordinates (*C_x_*, *C_y_*, *C_z_*) of the center of the atomic group that are taken, with no change, from the previous step.

### 4.3. Gradient with respect to the elements of the inertia matrix (Step *c*)   

Using the partial derivatives of *a_S_*, *b_S_*, *c_S_* [equation (50) in Appendix *C* [App appc]] and of the 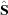 elements [equation (43) in Appendix *C* [App appc]] with respect to the elements of the matrix **S** [equation (13)], we obtain
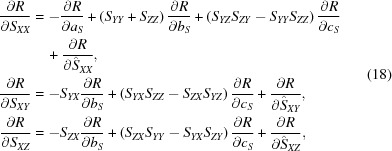
and similar expressions for the other six derivatives easily obtained from equation (18) by the corresponding cyclic substitution 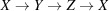 of the indices, *e.g.* to obtain 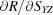 we make the following substitutions in the second equation: 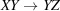, 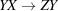, 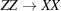, 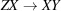, 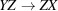.

### 4.4. Gradient with respect to the coordinates in the centered system (Step *b*)   

We obtain the derivatives with respect to the atomic coordinates, *k* = 1, …, *K*,
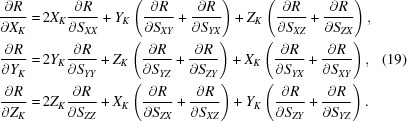



### 4.5. Gradient of restraint with respect to the original coordinates (Step *a*)   

Using the partial derivatives of equations (3) and (12) and the notation *W_j_* = 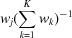,
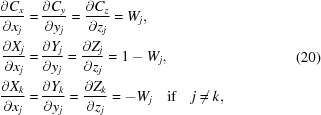
and knowing that all cross-term derivatives are equal to 0, we obtain the required gradient with respect to the original atomic coordinates 
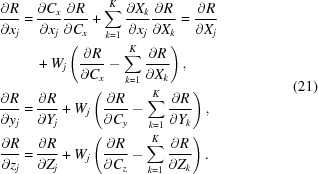



## 5. Implementation of parallelity restraints in *cctbx*   

### 5.1. *Computational Crystallography Toolbox*   

The *Computational Crystallography Toolbox* (*cctbx*) is an open-source library with numerous functionalities necessary to construct crystallography-oriented programs. In fact, it finds applications beyond crystallography, such as small-angle scattering (*sastbx*; Liu *et al.*, 2012 ▸) or cryo-EM (Afonine *et al.*, 2013 ▸). *cctbx* contains tools to handle various objects such as crystal symmetry, diffraction data, maps (crystallographic or cryo-EM) and atomic models, and provides an array of building blocks necessary to construct complex procedures such as model refinement (Afonine *et al.*, 2012 ▸) or diffraction data analysis (Sauter *et al.*, 2013 ▸; Parkhurst *et al.*, 2014 ▸). In particular, to support macromolecular refinement algorithms *cctbx* includes tools to handle various geometry restraints (Grosse-Kunstleve & Adams, 2004 ▸). The main set of geometry restraints includes those on covalent bonds and angles, chirality, dihedral (torsion) angles, non-bonded interactions, planar groups, and now parallelity. The overall restraint target, the sum in equation (1), is the sum of contributions from all restraint types
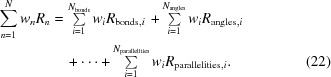
If additional information about the molecule or its environment is available and deemed necessary to use, additional terms can be added to equation (22) (Echols *et al.*, 2010 ▸; Headd *et al.*, 2012 ▸, 2014 ▸). Such additional information may be the regular conformations of a protein’s secondary structure, non-crystallographic symmetry or similarity to a previously solved structure. Technically, adding additional restraints means adding additional terms to equation (22), which in turn means that an algorithm for calculating the additional restraint target and its gradient with respect to atomic coordinates, and the data structures for holding information about the atoms involved, are required.

The implementation of the parallelity restraint in *cctbx* is very similar to other restraints (angle, dihedral angle, planarity *etc.*) and therefore may serve as an example of the common framework for restraints.

### 5.2. General approach to defining stereochemical restraints in *cctbx*   

The source code for commonly used restraints is located at  http://sourceforge.net/p/cctbx/code/HEAD/tree/trunk/cctbx/geometry_restraints in the files bond.h, bond_bpl.cpp, angle.h, angle_bpl.cpp, dihedral.h, dihedral_bpl.cpp, chirality.h, chirality_bpl.cpp, nonbonded.h, nonbonded_bpl.cpp, planarity.h, planarity_bpl.cpp, parallelity.h and parallelity_bpl.cpp.

Files with the name <type of restraint>_bpl.cpp contain boost.python (Abrahams & Grosse-Kunstleve, 2003 ▸) descriptions of classes and functions for the restraint, and the bpl in the file names stands for ‘boost.python library’. Generally, these files contain boost.python wrappers for classes describing the restraint and the associated data structure (termed proxy; see below) holding the information necessary to calculate the restraint. Files with the name <type of restraint>.h contain the actual implementation of classes and functions for the particular type of restraint.

### 5.3. Proxy class   

The calculation of a particular restraint requires information about the atoms involved, such as the atomic coordinates, and the restraint target and weight. Since the restraints are built once at the beginning of refinement, while the atomic coordinates change during refinement, information about the atoms that participate in a restraint and the actual atomic coordinates is decoupled. The data structure that holds the restraint information is called a proxy, and it contains integer indices that can be used to access a particular atom in an array of atoms and therefore individual coordinates, along with some other restraint-specific information.

For example, the bond proxy contains a pair of integer indices (atom numbers in an atom list), the restraint target value and weight, the slack and limit values, a flag that specifies which function to use (least-squares or top-out) and a rotation matrix operator in case bonded atoms belong to different crystallographic symmetry copies, and an indicator to distinguish the origin of a particular restraint (*e.g.* covalent or hydrogen bond). Since each proxy object contains all the parameters necessary to define a particular restraint, each restraint can be parameterized individually.

The parallelity proxy is organized in a very similar manner to the planarity proxy, the difference being that the atom numbers are split and contained in two groups because they define two separate planes with an arbitrary number of atoms in each one.

Usually, one constructs multiple restraints of one type for a structure, so it is convenient to have a structure to store an array of proxies of one type and perform various operations on them. Similarly to other restraint types, for the parallelity restraints there is an array that can contain multiple proxy objects, called the shared_parallelity_proxy. Operations on proxy arrays include selection (this allows selection of a subset of restraints corresponding to a part of the structure), deletion (restraints can be deleted for a part of structure), and calulation of root mean-square deviations of the model from target values, absolute differences between the restraint target and actual model values, the restraint target value, gradients and so on.

### 5.4. Class to handle parallelity restraint   

The parallelity restraint itself [equation (4)] and its modifications [equations (7) and (11)] are implemented as a C++ class parallelity available in Python *via* boost.python from cctbx.geometry_restraints.parallelity. It has two constructors. One requires providing all parameters to define a parallelity proxy (such as target value, type of function *etc.*) and two sets of Cartesian atomic coordinates corresponding to the two planes. The other constructor takes an array with Cartesian coordinates of atoms and an instance of the parallelity proxy class that contains the rest of the required information.

All constructors contain a call to the init_deltas() function which calculates all necessary values, such as the overall restraint value and the gradients. This means that instantiation of a parallelity object leads to the immediate calculation of the target value and gradients for the restraint.

To simplify the handling of the slack functionality and to make it universal for harmonic and top-out potentials, the conditions of equation (11) are evaluated first and θ_0_ adjusted accordingly before any other calculations. This makes it possible to avoid the gradient calculation if it is not necessary, for example when |θ − θ_0_| ≤ slack, and also to use all of the derivative equations for harmonic and top-out potentials without change.

In addition to the two constructors, the parallelity class has member functions residual() and gradients() that return the value of the restraint function and gradients with respect to the atomic coordinates. Since all necessary calculations are already performed in the constructor, these functions return precalculated values.

## 6. Discussion   

Applying the general principles of refinement program construction, it is straightforward to build various stereochemical restraints. However, a careful analysis is required to choose an appropriate function for the restraint target. This choice is based on a function’s ability to provide the desired behavior, both near the minimum and far from it. Possible singularities when calculating the target and its derivatives must also be taken into account. With a view to improving the refinement of atomic models, especially at low resolution, we have developed new restraints to control the angle between two approximately planar atomic groups. As a particular and more frequent case, this restraint imposes the groups to be parallel or near parallel. We have proposed a practical algorithm to calculate this target and its gradient. This algorithm has been implemented in the *cctbx* open-source library, making it available in *PHENIX* and to the broader crystallographic and macromolecular structure community. All calculation details are provided, making it easy to add other types of restraint in the future.

## Appendix A Other restraints on plane groups and their gradients

### A1. Planarity targets

This restraint requires an atomic group to be as planar as possible. Calculation of the target uses exactly the same Steps *a*–*e* described in §3 and Fig. 2 ▸ and differs only in Step *f*. As for the other steps, in order to avoid confusion below when calculating the gradients we introduce new parameters , ,  instead of the eigenvalues of the inertia matrix (Appendix *C* [App appc]).

(Step *f*) The simplest target can be expressed as (Urzhumtsev, 1991 ▸)

which takes the value of the minimal eigenvalue already calculated at Step *d*. Another approach used currently (Blanc & Paciorek, 2001 ▸) uses a different method of calculation but is essentially the same.

The restraint of equation (23) does not take into account the number of atoms in the restrained group. This leads to the effect that the more populated group (*i.e.* the group with more atoms) will be forced to be more planar than the less populated group given the same weight. This may be corrected by normalizing by the number of atoms in the group:

While equation (23) expresses a type of absolute value of the ‘flatness’ of the ellipsoid of inertia, other targets may estimate a relative value of such a flatness using several eigenvalues, for example

For this target the normalization with respect to the number of atoms is performed automatically.

### A2. Parallel-distance target

Another geometric restraint that can be imposed on atomic models, especially those of nucleic acids, is requiring the distance between two parallel planes to be equal to a prescribed value *l*_target_. Its calculation follows the same algorithm presented above. We first pass through the same Steps *a*–*e*, finishing as follows.

(Step *f*1) We repeat Step *f*1 of the parallelity restraint (§3.6) and calculate normalized eigenvectors.

(Step *f*2) We then calculate the median (subscript med) direction between two normal vectors,

(Step *f*3) and normalize it,

(Step *f*4) Finally, we calculate the ‘oriented’ distance between the best planes that pass through the two atomic groups as

(Step *f*5) and the target itself,

Target (29) uses the square of the parameter from equation (28) to remove the influence of the sign of the projection of (**C**_2_ − **C**_1_) on the unit vector **n**_med_.

## Appendix B Eigenvalues and eigenvectors of the inertia matrix

Below, when describing the intermediate transformations of parameters at Steps *d* and *e* of the principal algorithm used in our program, the output parameters of each transformation are the input parameters for the next one. We start from the following input parameters:

At Step *d*, all these parameters except *a_S_*, *b_S_* and *c_S_* remain unchanged and we omit them in the explicit list of parameters in this appendix.

(Step *d*1) We replace the initial equation (14) by

with the new parameters replacing *a_S_*, *b_S_* and *c_S_*

If the value of *g_S_* in equation (32) is close to zero, or even slightly negative because of rounding errors, we assign *g*_*S*_ = 0; when an atomic group is already more or less planar this will not be the case.

(Step *d*2) We calculate the cosine ξ_*S*_ of the characteristic angle 3γ_*S*_ as

resulting in the output parameters ,  and .

(Step *d*3) First, we calculate

and then

The output parameters are , , ,  and .

(Step *d*4) The eigenvalues are

where λ_+_ ≤ λ_−_ ≤ λ_0_. We then calculate the corresponding eigenvectors starting from the set of parameters

(Step *e*1) The eigenvector **N**_+_ corresponding to the minimal eigenvalue λ_+_ satisfies the condition

This means that it is orthogonal to the three vectors **t**_*X*_ = , **t**_*Y*_ = , **t**_*Z*_ = . In other words, **N**_+_ can be taken as the vector product of any two of them. To minimize rounding errors and avoid singular situations, for example when two chosen vectors are collinear or practically collinear to each other, we check all permutations

For each of these permutations we calculate the vector

and accept the permutation for which the norm of equation (40) is maximal.

(Step *e*2) After selecting vectors **t**_1_ and **t**_2_ we take the corresponding product of equation (40) as the eigenvector to define the ‘best plane’.

The calculation of other eigenvectors (if necessary) follows the same procedure. Currently known geometric restraints, including parallelity ones, do not require more than one eigenvector that corresponds to the minimal eigenvalue λ_+_. However, considering other possible new restraints, the complete transition from atomic coordinates to plane parameters in the general case should include as output all three eigenvalues and all three corresponding eigenvectors

## Appendix C Gradient with respect to the coefficients of the characteristic equation

Recalculation of the gradient is applied to each pseudo-planar group, one by one, and does not depend on the particular plane-group restraint *R*. The input to this computational module is a set of partial derivatives of *R* with respect to the center of the group, to the three eigenvalues and to the coordinates of the three eigenvectors, as mentioned above. For a particular restraint, some of these derivatives, if not most of them, may be equal to zero.

(Step *e*2) Derivation of the eigenvector with respect to the **t** vectors.

For the eigenvector **N** = **N**_+_ calculated by equation (40) we have

The derivatives ,  and  differ from equation (42) by swapping the indices 1 and 2 and inverting the sign.

(Step *e*1) Derivation of the eigenvector with respect to the eigenvalues and  elements.

The partial derivatives with respect to the  elements depend on the choice of vectors for **t**_1_ and **t**_2_ when calculating **N**, equation (40). For example, when (**t**_1_ = **t**_*X*_, **t**_2_ = **t**_*Y*_)

The equations are similar for other choices of **t**_1_ and **t**_2_. It is important not to forget the direct partial derivative  if the target depends directly on the eigenvalue. The partial derivatives of (43) with respect to other eigenvalues, for example when using targets such as equation (24), are calculated similarly. In this work we do not consider the targets depending on eigenvectors other than **N**_+_; equations (42) and (43) may easily be generalized for more complicated targets in the future, but here we avoid giving these simple but lengthy expressions.

(Step *d*4) Derivation of eigenvalues with respect to , , , , . For each eigenvalue we obtain

Partial derivatives with respect to the matrix  elements were calculated above [equation (43)].

(Step *d*3) Derivation of eigenvalues with respect to intermediate variables. Using the property of a derivative of the inverse function, we have

and similar equations for τ_*S*−_ and τ_*S*0_. Therefore

(Step *d*2) To obtain the derivatives with respect to *g_S_* and *h_S_*, we first calculate

with which

(Step *d*1) Derivation of eigenvalues with respect to the coefficients of the original characteristic equation. We can use

This results in

## Appendix D Calculation of the parallelity targets and their gradients with respect to the normal vectors

### d1. Parallelity targets

Let each of the two pseudo-planar atomic groups be described by the Cartesian coordinates of the vector normal to its respective best plane (**N**_1_ and **N**_2_), not necessarily of unit length.

(Step *f*1) First we calculate the unit normal vectors as

The correct sign for σ must be chosen so that the absolute value of the angle θ between the normal vectors is not larger than π/2.

(Step *f*2) Once the normalized vectors are known, we calculate the cosine and sine of the angle θ between them, considering that sinθ ≥ 0 because 0 ≤ θ ≤ π/2:

(Step *f*3) Finally, we calculate the value of the chosen target. Generally, one needs first to calculate the intermediate parameters

which lead to the targets of equations (4), (7) and (8) as

Equation (10) for *n* > 2 may require, for example, using the De Moivre formula (Lial *et al.*, 2008 ▸) *C*_*nD*θ_ = Re(*C*_*D*θ_ + *iS*_*D*θ_)^*n*^; we do not provide these expressions here.

Obviously, the particular and most frequent case θ_0_ = 0 can be considered as an independent target

which does not require equations (53)–(55).

### d2. Gradient with respect to the normal vectors

(Step *f*3) Inversion of the last step of the target calculation provides the values of the gradients of the targets of equations (4), (7) and (8) with respect to the intermediate parameters as

Then, using the chain rule,

(Step *f*2) At the next step, applying the chain rule, we obtain

and similarly

The expressions for ,  and  are similar to equations (64) and (65) with *n*_1*X*_, *n*_1*Y*_, *n*_1*Z*_ instead of *n*_2*X*_, *n*_2*Y*_, *n*_2*Z*_ in the right-hand parts and with a − sign instead of a + before the parentheses.

(Step *f*1) Taking the values obtained in equations (64) and (65), we calculate

where ||**N**_1_|| = (**N**_1_·**N**_1_)^1/2^. In the same way, we obtain

Expressions for ,  and  are obtained from equations (66)–(68) by substituting σ||**N**_2_||^−3^ for ||**N**_1_||^−3^ and replacing the index 1 by 2 for the rest.

## References

[bb1] Abrahams, D. & Grosse-Kunstleve, R. W. (2003). *C/C++ Users J.* **21**, 29–36.

[bb25] Adams *et al.* (2010). *Acta Cryst.* D**66**, 213–221.

[bb2] Afonine, P. V., Grosse-Kunstleve, R. W., Echols, N., Headd, J. J., Moriarty, N. W., Mustyakimov, M., Terwilliger, T. C., Urzhumtsev, A., Zwart, P. H. & Adams, P. D. (2012). *Acta Cryst.* D**68**, 352–367. 10.1107/S0907444912001308 PMC3322595 22505256

[bb3] Afonine, P. V., Headd, J. J., Terwilliger, T. C. & Adams, P. D. (2013). *Comput. Crystallogr. Newsl.* **4**, 43–44.

[bb4] Afonine, P. V. & Urzhumtsev, A. (2004). *Acta Cryst.* A**60**, 19–32. 10.1107/s0108767303022062 14691324

[bb5] Baur, W. & Strassen, V. (1983). *Theor. Comput. Sci.* **22**, 317–330.

[bb6] Blanc, E. & Paciorek, W. (2001). *J. Appl. Cryst.* **34**, 480–483.

[bb7] Brown, A., Long, F., Nicholls, R. A., Toots, J., Emsley, P. & Murshudov, G. (2015). *Acta Cryst.* D**71**, 136–153. 10.1107/S1399004714021683 PMC4304694 25615868

[bb8] Dennis, J. E. Jr & Welsch, R. E. (1987). *Commun. Stat. Simul. Comput.* **7**, 345–359.

[bb9] Echols, N., Headd, J. J., Afonine, P. & Adams, P. D. (2010). *Comput. Crystallogr. Newsl.* **1**, 12–17.

[bb11] Grosse-Kunstleve, R. W. & Adams, P. D. (2004). *IUCr Comput. Commission. Newsl.* **4**, 19–36.

[bb12] Grosse-Kunstleve, R. W., Sauter, N. K., Moriarty, N. W. & Adams, P. D. (2002). *J. Appl. Cryst.* **35**, 126–136.

[bb13] Headd, J. J., Echols, N., Afonine, P. V., Grosse-Kunstleve, R. W., Chen, V. B., Moriarty, N. W., Richardson, D. C., Richardson, J. S. & Adams, P. D. (2012). *Acta Cryst.* D**68**, 381–390. 10.1107/S0907444911047834 PMC3322597 22505258

[bb26] Headd, J. J., Echols, N., Afonine, P. V., Moriarty, N. W., Gildea. R. J. & Adams, P. D. (2014). *Acta Cryst.* D**70**, 1346–1356. 10.1107/S1399004714003277 PMC4014122 24816103

[bb14] Jack, A. & Levitt, M. (1978). *Acta Cryst.* A**34**, 931–935.

[bb15] Kim, K. M., Nesterov, Yu. E. & Cherkassky, B. V. (1984). *Dokl. Acad. Nauk. SSSR*, **275**, 1306–1309.

[bb16] Konnert, J. H. (1976). *Acta Cryst.* A**32**, 614–617.

[bb17] Lial, M. L., Hornsby, J., Schneider, D. I., Callie, J. & Daniels (2008). *College Algebra and Trigonometry*, 4th ed., p.792. Boston: Pearson/Addison Wesley.

[bb18] Liu, H., Hexemer, A. & Zwart, P. H. (2012). *J. Appl. Cryst.* **45**, 587–593.

[bb19] Lunin, V. Yu. & Urzhumtsev, A. G. (1985). *Acta Cryst.* A**41**, 327–333.

[bb20] Murshudov, G. N., Skubák, P., Lebedev, A. A., Pannu, N. S., Steiner, R. A., Nicholls, R. A., Winn, M. D., Long, F. & Vagin, A. A. (2011). *Acta Cryst.* D**67**, 355–367. 10.1107/S0907444911001314 PMC3069751 21460454

[bb21] Parkhurst, J. M., Brewster, A. S., Fuentes-Montero, L., Waterman, D. G., Hattne, J., Ashton, A. W., Echols, N., Evans, G., Sauter, N. K. & Winter, G. (2014). *J. Appl. Cryst.* **47**, 1459–1465. 10.1107/S1600576714011996 PMC4119952 25242914

[bb22] Sauter, N. K., Hattne, J., Grosse-Kunstleve, R. W. & Echols, N. (2013). *Acta Cryst.* D**69**, 1274–1282. 10.1107/S0907444913000863 PMC3689530 23793153

[bb23] Smart, O. S., Womack, T. O., Flensburg, C., Keller, P., Paciorek, W., Sharff, A., Vonrhein, C. & Bricogne, G. (2012). *Acta Cryst.* D**68**, 368–380. 10.1107/S0907444911056058 PMC3322596 22505257

[bb24] Urzhumtsev, A. G. (1991). *Acta Cryst.* A**47**, 723–727.

